# Volatile organic compounds exposure associated with frailty in United States adults from NHANES 2011–2018

**DOI:** 10.3389/fpubh.2025.1655214

**Published:** 2025-09-16

**Authors:** Tian-Zhen Qu, Tian Zhang, Qing-Yun Huang, Xing-Lan Chen, Ye Zhu

**Affiliations:** ^1^Department of General Practice, Jiangyin People's Hospital, Wuxi, China; ^2^Department of Clinical Medicine, Nanjing Medical University, Nanjing, China; ^3^Department of Gerontology, Geriatric Hospital Affiliated to Nanjing Medical University, Nanjing, China; ^4^Department of Medical Laboratory, Wuxi Huishan District Center for Disease Control and Prevention, Wuxi, China

**Keywords:** volatile organic compounds, frailty, oxidative stress, mixture modeling, weighted quantile sum, NHANES

## Abstract

**Background:**

Whether environmentally relevant exposure to volatile organic compounds (VOCs) contributes to frailty remains unknown. We examined urinary VOC metabolites (VOCms) and their mixtures in relation to frailty in a nationally representative U.S. cohort.

**Methods:**

We analysed 2,715 adults (≥ 20 y) from NHANES 2011–2018 in a cross-sectional design. Frailty was defined with a 48-item index. Sixteen creatinine-adjusted VOCms were quantified. Single metabolites were evaluated with survey-weighted logistic regression. Two-directional weighted-quantile-sum regression (WQS), grouped Bayesian kernel machine regression (BKMR) and quantile g-computation (qgcomp) characterized mixture effects, and sex- and age-stratified subgroup analyses were performed. Mediation by *γ*-glutamyl-transferase (GGT), bilirubin, albumin, the Dietary Oxidant/Antioxidant Balance Score (OBS), and high-sensitivity C-reactive protein (hs-CRP) was assessed.

**Results:**

Four metabolites—DHBMA, CEMA, HPMMA and MHBMA3—were each positively associated with frailty (adjusted OR per log₁₀-unit 1.67–2.59). The positive WQS index increased frailty odds by 25% (OR = 1.25, 95% CI 1.17–1.33), whereas the negative index lowered odds by 17% (OR = 0.83, 0.75–0.91). Only the positive index remained significant in men and in adults ≥ 65 y; MHBMA3 dominated male weights (18%), HPMMA female weights (16%). BKMR confirmed a monotonic dose–response for the positive group, whereas qgcomp detected no overall effect. Bilirubin and albumin jointly mediated 5–20% of the associations; GGT showed no significant mediation.

**Conclusion:**

Urinary VOCm mixtures are linked to frailty at population exposure levels, with risk driven by four metabolites and most pronounced in men and older adults. Oxidative stress explains part—but not all—of the association, suggesting additional pathways. Reducing VOC exposure may help preserve physiological reserve; longitudinal studies are warranted to confirm causality.

## 1 Introduction

Volatile organic compounds (VOCs) are a diverse class of gases emitted from fuels, plastics, solvents, and tobacco smoke; once absorbed, many are conjugated with cysteine and excreted as mercapturic-acid metabolites of VOCs (VOCms), providing an objective record of internal dose (1, 2). After absorption, VOCs undergo sequential biotransformation through Phase I and Phase II enzymatic pathways. Cytochrome P450-mediated oxidation generates electrophilic intermediates, which conjugate with glutathione and are subsequently processed via the mercapturic acid pathway. The resulting N-acetyl-L-cysteine conjugates are excreted in urine as mercapturic acid metabolites, which serve as stable and specific biomarkers of VOC exposure.

These pollutants are virtually ubiquitous: more than 90% of U.S. adults carry detectable urinary levels of at least one VOCm (3). Epidemiologic studies have linked elevated VOC or VOCm burdens to a spectrum of age-related disorders, including cardiovascular disease, liver steatosis, rheumatoid arthritis, and sarcopenia (4, 5). Mixture-oriented analyses show that combined exposure to N-acetyl-S-(3,4-dihydroxybutyl)-L-cysteine (DHBMA, a urinary metabolite of 1,3-butadiene), N-acetyl-S-(2-Carboxyethyl)-L-cysteine (CEMA, a urinary metabolite of acrylonitrile), and several aliphatic metabolites amplifies cardiometabolic risk beyond single-compound models. Mechanistic data implicate oxidative stress, endothelial dysfunction, and mitochondrial impairment (6, 7). Oxidative stress, in particular, is increasingly recognized as a molecular catalyst of frailty, promoting muscle loss, inflammation, and metabolic dysregulation across the adult life-course (8). Because adults accumulate lifelong exposure while detoxification capacity declines with age, the entire adult life-course—particularly later life—may be vulnerable to subtle shifts in VOC mixture profiles that translate into clinically meaningful health deficits.

Frailty, a multidimensional syndrome of diminished physiologic reserve, has emerged as an overarching indicator of healthy ageing, predicting disability, hospitalization, and mortality better than single-organ metrics (9). Although U.S. prevalence reaches 10–15% among adults ≥ 65 years and exceeds 25% past age 80, frailty can begin much earlier and progress across adulthood (10). While intrinsic factors such as sarcopenia, inflammation, and endocrine change initiate the frailty trajectory, mounting evidence suggests that environmental pollution accelerates its progression. Meta-analytic data show that each 10 μg m^−3^ rise in fine particulate matter with an aerodynamic diameter ≤2.5 μm (PM₂․₅) increases frailty odds by ~19%, and cohort studies in China and India link indoor or household air pollution to faster frailty development (11–13). Yet these investigations focus on particulate and gaseous pollutants; the toxicologically diverse VOC family remains largely unexplored, making the VOC–frailty nexus both biologically plausible and clinically urgent.

In the past five years, nationally representative NHANES analyses and large Asian cohorts have mapped the health footprint of urinary VOC metabolites. Two NHANES studies tie urinary VOCms to ageing outcomes: elevated DHBMA and CEMA, together with N-acetyl-S-(3-hydroxypropyl-1-methyl)-L-cysteine (HPMMA), predicted 30–60% higher sarcopenia odds, while a mixture dominated by N-acetyl-S-(2-cyanoethyl)-L-cysteine (CYMA) was inversely related to the anti-ageing protein *α*-Klotho (14, 15). Mixture-model analyses indicate that combined VOCm exposure aggravates blood-pressure control, elevates the odds of metabolic syndrome and type 2 diabetes, and increases cardiovascular-disease risk by roughly 20% (4, 16, 17). Because hypertension, diabetes, coronary heart disease, sarcopenia, and low *α*-Klotho are all recognized precursors or integral components of the frailty trajectory, this constellation of cardiovascular, metabolic, and musculoskeletal disturbances offers a compelling mechanistic bridge linking VOCm mixtures to frailty.

Pollution-frailty research has grown in parallel. A nine-study meta-analysis reported a 19% increase in frailty odds per 10 μg m^−3^ PM₂․₅ (11), while Chinese and Indian cohorts linked indoor pollutants to accelerated frailty progression (12, 13). Collectively, the evidence highlights two themes: first, chemical mixtures often produce stronger—and sometimes opposing—health effects than individual pollutants; second, susceptibility to these exposures rises across adulthood and is greatest in later life. Nevertheless, no investigation has yet examined whether urinary VOCms—alone or in combination—are associated with frailty in the general adult population, leaving a critical gap at the intersection of geriatric epidemiology and exposomics. It also remains unclear which structural classes of VOCms drive risk, whether opposing mixture effects coexist, and to what extent oxidative stress mediates any association.

We therefore assessed urinary VOCm mixtures and frailty in U.S. adults aged ≥ 20 years using NHANES 2011–2018. Positive and negative mixture effects were estimated with two-directional weighted-quantile-sum regression and verified with quantile g-computation. Key metabolites were then identified by LASSO penalized selection and examined in direction-specific Bayesian kernel machine regression to delineate dose–response functions. Finally, mediation analysis evaluated *γ*-glutamyl-transferase, bilirubin, and albumin as oxidative-stress pathways, providing both risk estimates and mechanistic insight.

## 2 Methods

### 2.1 Population

**Figure 1:**
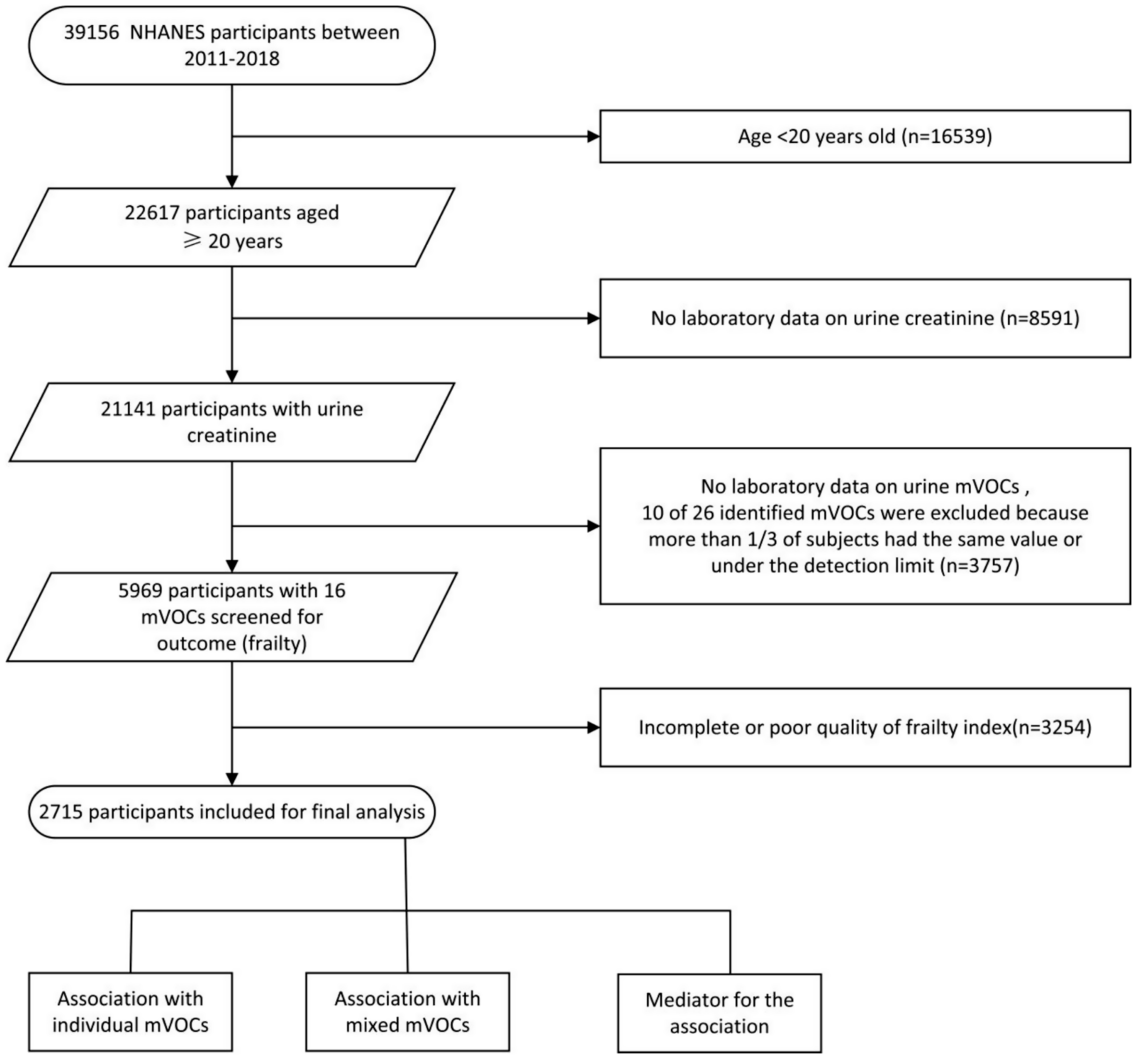
Participant selection flowchart.

From four NHANES cycles (2011–2018, *n* = 39,156), we retained adults aged ≥ 20 years (*n* = 22,617) and removed 8,591 participants without urine-creatinine data, leaving 14,026 adults. Restricting the sample to those with complete measurements for 16 reliably detectable VOCm metabolites—together with their parent VOCs listed in Table 1—excluded another 3,757 participants. After omitting 3,254 individuals with incomplete frailty data, the final analytic cohort comprised 2,715 adults for single-metabolite, mixture, and mediation analyses. The full selection process is shown in Figure 1.

**Table 1:** Relative parent volatile organic compounds (VOCs) to the metabolites of VOCs (VOCms).

Parent VOCs	Urine metabolites of VOCs (VOCms)	Abbreviation of VOCms	LLOD (ng/mL)
Acrolein	N-Acetyl-S-(3-hydroxypropyl)-L-cysteine	3HPMA	13.00
Crotonaldehyde	N-Acetyl-S-(3-hydroxypropyl-1-methyl)-L-cysteine	HPMMA	1.13
Acrylamide	N-Acetyl-S-(2-carbamoylethyl)-L-cysteine	AAMA	2.20
Acrylonitrile	N-Acetyl-S-(2-Carbxyethyl)-L-cysteine	CEMA	6.96
	N-Acetyl-S-(2-cyanoethyl)-L-cysteine	CYMA	0.50
Cyanide	2-Aminothiazoline-4-carboxylic acid	ATCA	15.00
Ethylbenzene, styrene	Phenylglyoxylic acid	PGA	12.00
Benzene	Mandelic acid	MA	12.00
N, N-Dimethylformamide	N-Acetyl-S-(N-methylcarbamoyl)-L-cysteine	AMCC	6.26
Propylene oxide	N-Acetyl-S-(2-hydroxypropyl)-L-cysteine	2HPMA	5.30
Styrene	N-Acetyl-S-(benzyl)-L-cysteine	SBMA	0.50
Xylene	2-Methylhippuric acid	2MHA	5.00
	3,4-Methylhippuric acid	3,4-MHA	8.00
1,3-Butadiene	N-Acetyl-S-(4-hydroxy-2-butenyl)-L-cysteine	MHBMA3	0.60
	N-ace-S- (3,4-dihidxybutl)-L-cysteine	DHBMA	5.25
1-Bromopropane	N-acetyl-S-(n-propyl)-L-cysteine	BPMA	1.20

LLOD, lower limit of detection.

### 2.2 Exposure

Participants provided non-fasting urine samples, stored at −70 °C until analysis. Urinary VOCms were quantified by ultra-performance liquid chromatography–tandem mass spectrometry using stable isotope-labeled internal standards. We emphasize that all laboratory analyses were conducted by the National Center for Environmental Health, Centers for Disease Control and Prevention (CDC) as part of the NHANES biomonitoring program. The LC–MS/MS workflow, including sample preparation, chromatographic separation, mass spectrometry conditions, and the use of isotope-labeled internal standards, followed standardized CDC laboratory protocols, which are publicly available in the NHANES Laboratory Method Files. The analyzed VOC metabolites included 2-methylhippuric acid (2-MHA), 3,4-methylhippuric acid (3,4-MHA), N-Acetyl-S-(2-carbamoylethyl)-L-Cysteine (AAMA), N-Acetyl-S-(N-methylcarbamoyl)-L-Cysteine (AMCC), 2-amino-thiazoline-4-carboxylic acid (ATCA), N-Acetyl-S-(benzyl)-L-Cysteine (SBMA), N-Acetyl-S-(n-propyl)-L-Cysteine (BPMA), mandelic acid (MA), N-Acetyl-S- (4-hydroxy-2-butenyl)-L-Cysteine (MHBMA3), phenylglyoxylic acid (PGA), as well as DHBMA, CEMA, HPMMA, and CYMA. In accordance with established practices in NHANES VOC biomonitoring research, we excluded VOCms for which more than one-third (>33%) of participants had concentrations below the analytical limit of detection (LOD) or had identical values across all participants. This threshold is commonly applied to minimize bias from heavily censored data and to ensure sufficient variability for reliable statistical modeling (18, 19). Concentrations below the limit of detection (LOD) were imputed as LOD/√2, following NHANES analytic guidelines (20).

### 2.3 Covariates

We adjusted for a set of demographic, socioeconomic, and lifestyle variables that have been consistently associated with both VOC exposure and frailty in prior epidemiological research (21, 22). Age and sex were included as fundamental demographic factors. Race/ethnicity was categorized as non-Hispanic White, non-Hispanic Black, non-Hispanic Asian, Mexican American, other Hispanic, or other race, reflecting NHANES classification standards. Educational attainment was grouped into three levels: less than 11th grade, high-school graduate, and at least some college, to capture differences in socioeconomic background and health literacy. Smoking status (never, former, current) and physical activity level (none, moderate, vigorous) were derived from standardized NHANES questionnaires, as these behaviors influence both exposure patterns and frailty risk. Economic status was expressed as the family income-to-poverty ratio, divided into quartiles, while dietary intake was represented by total energy intake (mean of two 24-h recalls), also divided into quartiles; these variables account for nutritional and economic determinants of health. To control for temporal variation in sampling and laboratory procedures, we additionally adjusted for the NHANES survey cycle. Because BMI is included as one of the deficit items used to calculate the frailty index (Supplementary Table 1, item 42), it was not entered as a separate covariate in the primary models to avoid potential over-adjustment. In addition, a sensitivity analysis was performed by further adjusting for BMI. Missing values for covariates were imputed using the k-nearest neighbors (KNN) algorithm to ensure a complete dataset for analysis.

### 2.4 Outcome

Frailty was assessed with the 48-item NHANES Frailty Index described in earlier studies (23, 24) (item list in Supplementary Table 1). Participants were included if they had completed at least 80% of the items (≥39/48). For each participant, the index was calculated as the proportion of deficits present out of the total number of non-missing items, thereby yielding a score between 0 and 1. Following previous literature, scores <0.21 were classified as non-frailty and scores ≥0.21 as frailty (25, 26).

### 2.5 Statistical analysis

Missing covariates were imputed with KNN using the VIM package, preserving multivariable structure; family income showed the highest missingness (10.9%).

Baseline characteristics were analyzed using survey-weighted methods to account for the NHANES complex sampling design. Continuous variables were reported as medians with interquartile ranges (IQR) and compared using the Mann-Whitney U test, while categorical variables were presented as survey-weighted percentages and compared using the Rao–Scott adjusted χ^2^ test. All analyses incorporated NHANES sampling weights, strata, and primary sampling units (PSUs) to ensure nationally representative estimates.

Urinary VOCm concentrations were adjusted for urinary creatinine and log-transformed to normalize their distributions. These transformed VOCm levels were entered into survey-weighted logistic regression models both as continuous variables and as quartiles (with Q1 as the reference group) to evaluate their associations with frailty.

To assess mixture effects, we applied direction-specific Weighted Quantile Sum (WQS) regression. Urinary VOCms were categorized into quartiles to reduce skewness and limit the influence of extreme values. Separate positive and negative WQS indices were modeled, with each metabolite assigned a weight reflecting its relative contribution to the mixture effect (27, 28). In typical applications, a larger proportion of the dataset is used for training and a smaller part for validation. However, in this study, we chose a 40% training and 60% validation split because a larger validation set provided more stable weight estimation across repeated bootstrap samples, while the 40% training set was still sufficient to build the model given our relatively large sample size. For the primary WQS analyses, all measured VOC metabolites were included *a priori*, consistent with standard practice in WQS mixture modeling. This allows estimation of the overall mixture effect under directional constraints without preselection of exposures. To further assess robustness, we additionally performed a sensitivity analysis where LASSO-selected metabolites were entered into the WQS model. Each model was fitted with 1,000 bootstrap resamples using the gWQS R package.

We further performed quantile-based g-computation (qgcomp), a recently developed approach for mixture analysis that simultaneously estimates the overall effect of increasing all exposures by one quantile while allowing both positive and negative contributions of individual components. This method was implemented using the qgcomp R package, following the framework described by Keil et al. (29, 30). Results were reported as the overall mixture effect (per one quantile increase in the joint exposure index) with 95% confidence intervals, along with component-specific weights reflecting the relative positive or negative contributions of each VOC metabolite. Employing both WQS and qgcomp in parallel strengthened the interpretability and robustness of our mixture analysis findings.

Complementing the WQS and qgcomp analyses, we used Bayesian Kernel Machine Regression (BKMR) to further examine potential non-linear and interactive effects among VOCms. To improve computational efficiency and minimize noise from variables with negligible associations, we first applied the least absolute shrinkage and selection operator (LASSO) regression to the full set of urinary VOC metabolites. The subset of VOCs retained by LASSO was then entered into the BKMR models. Metabolites selected by LASSO were grouped according to the direction of their crude association with frailty (positive or negative) and analyzed in separate BKMR models.

BKMR models provided estimates of overall mixture effects, subgroup-specific effects for positively and negatively associated metabolites, and variable importance, expressed as group posterior inclusion probabilities (groupPIP) and conditional posterior inclusion probabilities (condPIP). Each model was run for 10,000 Markov Chain Monte Carlo (MCMC) iterations to ensure convergence, with stability verified through inspection of trace plots and convergence diagnostics (31, 32). All three mixture models (WQS, qgcomp, and BKMR) were adjusted for the same covariates as the primary logistic regression analyses, ensuring comparability across methods.

To explore potential effect modification, stratified analyses were conducted by sex (men vs. women) and by age group (< 65 years vs. ≥ 65 years). In each subgroup, WQS, qgcomp, and BKMR models were re-run using the same covariate adjustments as in the primary analyses. Stratified analyses were also stratified by smoking status (current, former, and never smokers), with WQS and BKMR models re-estimated using the same covariate adjustments as in the main analyses.

Additionally, to further explore potential synergistic and antagonistic effects within the mixture, we calculated the urinary DHBMA/SBMA concentration ratio (after creatinine standardization and log-transformation) and evaluated its association with frailty using survey-weighted logistic regression models. The ratio was analyzed both as a continuous variable and by quartiles.

Recognizing oxidative stress as a putative molecular catalyst of frailty (8), we examined whether redox biomarkers mediated the association between urinary VOCm mixtures and frailty. Mediation analyses were conducted with the mediation R package, using the WQS-derived mixture index as the exposure and frailty status as the outcome. Separate models were fitted for *γ*-glutamyl-transferase (GGT), bilirubin, and albumin, followed by a joint multiple-mediator model that included all three biomarkers. In addition, we extended the mediation framework to include the Dietary Antioxidant/Oxidant Balance Score (OBS), which reflects dietary oxidative balance, as an additional mediator. Given the limited availability of inflammatory markers in NHANES, we further conducted exploratory mediation analyses using high-sensitivity C-reactive protein (hs-CRP) as an inflammatory biomarker. Since hs-CRP was only measured in the 2015–2018 NHANES cycle, these analyses were restricted to that subsample. The oxidative balance score (OBS) was calculated based on 16 dietary components and 4 lifestyle factors (physical activity, body mass index, alcohol intake, and cotinine), following established protocols (33, 34). Antioxidants and prooxidants were scored in opposite directions, with higher OBS values reflecting a predominance of antioxidant exposures (see Supplementary Table 2).

Each analysis decomposed the total effect (TE) into a direct effect (DE) and an indirect effect (IE) operating through the mediator(s); the proportion mediated was calculated as IE/TE. Statistical inference relied on non-parametric bootstrapping with 1,000 resamples to generate survey-weighted 95% confidence intervals for DE, IE, and TE. All mediation models adjusted for the same covariates used in the primary analyses. All analyses were conducted in R (version 4.4.1), and statistical significance was defined as a two-sided *p* < 0.05.

### 2.6 Ethical approval

The NHANES survey protocol was approved by the National Center for Health Statistics Research Ethics Review Board, and written informed consent was obtained from all participants in accordance with the Declaration of Helsinki.

## 3 Results

### 3.1 Baseline characteristics

As shown in Table 2, frail adults (*n* = 1,383) were similar in age to non-frail adults (median 64 y) but were more often female, more likely to be non-Hispanic Black or of another minority race, and tended to have lower education and income (all *p* < 0.001). They reported less vigorous activity and more inactivity, and current smoking was slightly more common. Energy intake also shifted toward lower quartiles in the frailty group. Baseline characteristics after imputation of missing covariate values using the k-nearest neighbors (KNN) algorithm are provided in Supplementary Table 3, which shows patterns consistent with those in the primary dataset.

**Table 2:** Characteristics of participants.

Characteristics	Total	Non-frailty	Frailty	*p*-value
No. of participants	2,715	1,332	1,383	
Age (years)	64 (56–72)	64 (60–71)	64 (54–74)	0.637
Sex, %				<0.001
Female	1,322 (48.69%)	556 (41.74%)	766 (55.39%)	
Male	1,393 (51.31%)	776 (58.26%)	617 (44.61%)	
Ethnicity, %				<0.001
Mexican American	300 (11.05%)	158 (11.86%)	142 (10.27%)	
Other Hispanic	282 (10.39%)	140 (10.51%)	142 (10.27%)	
Non-Hispanic White	1,130 (41.62%)	532 (39.94%)	598 (43.24%)	
Non-Hispanic Black	656 (24.16%)	298 (22.37%)	358 (25.89%)	
Non-Hispanic Asian	245 (9.02%)	169 (12.69%)	76 (5.50%)	
Other Race	102 (3.76%)	35 (2.63%)	67 (4.84%)	
Education level %				<0.001
Less than high school	363 (13.38%)	159 (11.95%)	204 (14.75%)	
High school or GED	1,056 (38.92%)	479 (36.02%)	577 (41.72%)	
Above high school	1,294 (47.70%)	692 (52.03%)	602 (43.53%)	
(Missing)	2	2	0	
Physical activity				<0.001
Never	880 (32.68%)	320 (24.13%)	560 (40.97%)	
Moderate	399 (14.82%)	183 (13.80%)	216 (15.80%)	
Vigorous	1,414 (52.50%)	823 (62.07%)	591 (43.23%)	
(Missing)	22	6	16	
Smoking status				<0.001
Never	1,274 (46.94%)	704 (52.89%)	570 (41.21%)	
Former	568 (20.93%)	217 (16.30%)	351 (25.38%)	
Current	872 (32.13%)	410 (30.80%)	462 (33.41%)	
(Missing)	1	1	0	
Family income, %				<0.001
Q1 (<1.020)	605 (25.02%)	220 (18.58%)	385 (31.20%)	
Q2 (1.020–1.770)	607 (25.10%)	268 (22.64%)	339 (27.47%)	
Q3 (1.770–3.525)	601 (24.86%)	312 (26.35%)	289 (23.42%)	
Q4 (>3.525)	605 (25.02%)	384 (32.43%)	221 (17.91%)	
(Missing)	297	148	149	
Total energy intake(kcal)				0.005
Q1 (<1,362)	632 (25.01%)	278 (22.38%)	354 (27.55%)	
Q2 (1362–1812)	632 (25.01%)	301 (24.24%)	331 (25.76%)	
Q3 (1812–2,355)	633 (25.05%)	330 (26.57%)	303 (23.58%)	
Q4 (>2,355)	630 (24.93%)	333 (26.81%)	297 (23.11%)	
(Missing)	188	90	98	

The continuous variables were presented as median (interquartile range, IQR), and the categorical variables were presented as number and percentages.

### 3.2 Individual VOCms and frailty risk

In the non-adjusted multivariable regression models (Supplementary Figure 1), several urinary VOC metabolites showed significant positive associations with frailty. The strongest association was observed for DHBMA (OR = 5.85, 95% CI 3.12–10.98, *p* < 0.001). CEMA (OR = 2.60, 95% CI 1.94–3.49, *p* < 0.001) and HPMMA (OR = 2.40, 95% CI 1.81–3.18, *p* < 0.001) also demonstrated robust associations. Additional significant metabolites included AAMA (OR = 1.92, 95% CI 1.37–2.70, *p* < 0.001), AMCC (OR = 2.01, 95% CI 1.48–2.72, *p* < 0.001), CYMA (OR = 1.42, 95% CI 1.26–1.61, *p* < 0.001), MA (OR = 2.63, 95% CI 1.61–4.30, *p* < 0.001), and 3HPMA (OR = 1.88, 95% CI 1.45–2.45, *p* < 0.001).

**Figure 2:**
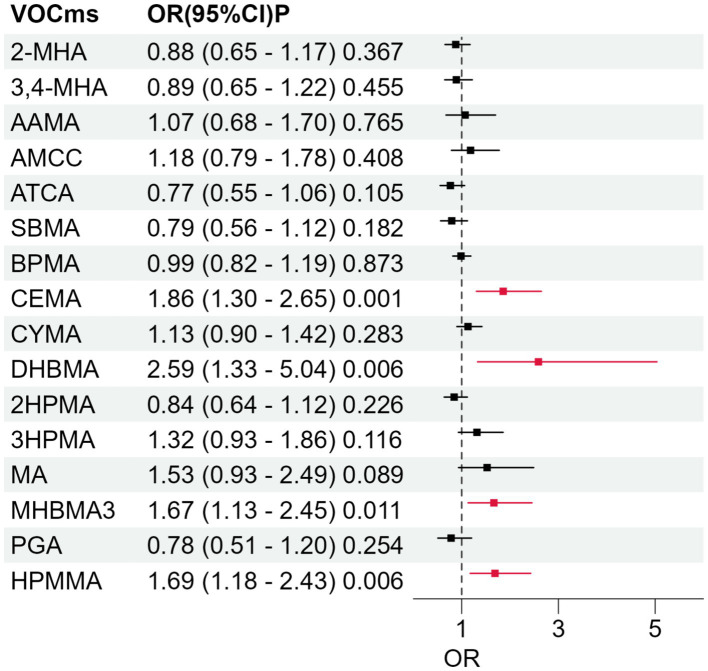
Adjusted associations between individual urinary VOCms and frailty risk. Forest plot displays survey-weighted odds ratios (ORs) and 95% confidence intervals (horizontal bars) obtained from multivariable logistic regression models. Each model was adjusted for sex, age, educational attainment, race/ethnicity, poverty-income ratio, smoking status, physical activity, survey cycle,and total energy intake. The dashed vertical line marks the null value (OR = 1). Red squares highlight metabolites with statistically significant associations (two-sided *p* < 0.05); black squares indicate non-significant findings.

We evaluated 16 urinary VOCm metabolites in relation to frailty using survey-weighted, fully adjusted logistic models (Figure 2). Four biomarkers showed robust, positive associations (*p* < 0.05). The strongest signal was for DHBMA (OR = 2.59, 95% CI 1.33–5.04), followed by CEMA (OR = 1.86, 95% CI 1.30–2.65). Both MHBMA3 (OR = 1.67, 95% CI 1.13–2.45) and HPMMA (OR = 1.69, 95% CI 1.18–2.43) were likewise associated with greater odds of frailty. As a sensitivity analysis, we further adjusted the models for BMI. The results were consistent with those of the primary models, suggesting that our findings were robust (Supplementary Figure 2).

Supplementary Figure 3 displays fully adjusted, quartile-based dose-response curves for the 16 urinary VOCms. For CEMA, MHBMA3, and HPMMA, frailty odds were higher in Q2–Q4 than in Q1, though without a strictly monotonic trend; for DHBMA, the association was evident only in Q3 and Q4. PGA shows a lone protective dip at Q3, whereas 3HPMA peaks at Q3—the only quartile with a significant risk elevation. The p for trend analysis indicated that CEMA, DHBMA, 3HPMA, MHBMA3, and HPMMA exhibited significant linear trends with frailty risk (all *p* < 0.05), while no significant trends were observed for other metabolites.

### 3.3 Mixed VOCms and frailty risk

#### 3.3.1 WQS and Qgcomp analysis

**Figure 3:**
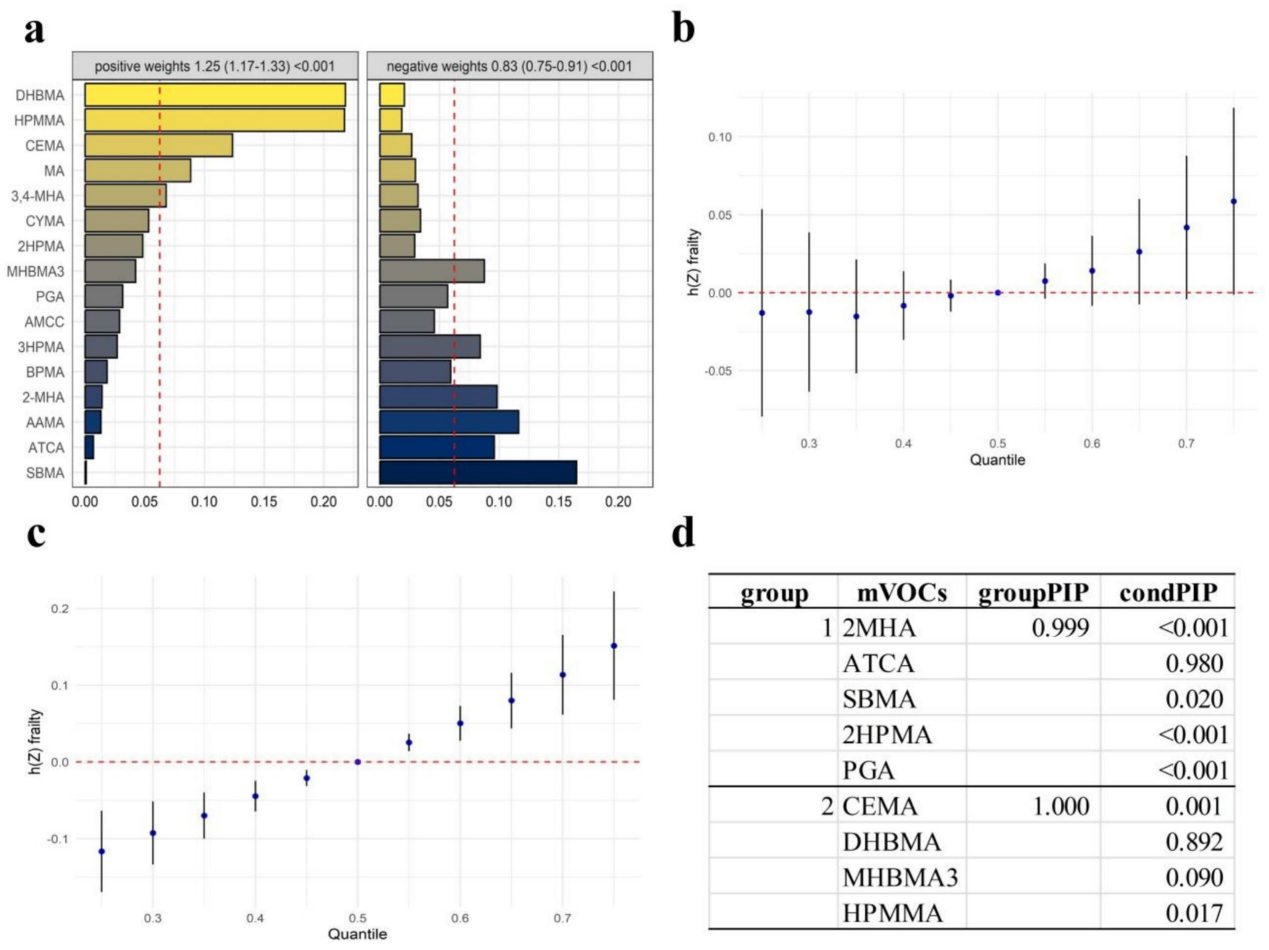
Integrated mixture analysis of urinary VOCms in relation to frailty. **(a)**Two-directional WQS weights. Bars show positive-(left) and negative-weight (right) contributions to the frailty mixture; red dashed lines mark the equal-weight threshold. Mixture odds ratios (OR, 95% CI) appear above each panel; **(b)** BKMR—overall mixture. Posterior mean frailty function h (Z) across exposure quantiles for the full set of nine LASSO-selected VOCms (shaded bars = 95% credible intervals; red dashed line = null); **(c)** BKMR—positively associated group. Exposure-response curve for the four VOCms with positive LASSO coefficients; **(d)** Hierarchical BKMR selection. Posterior inclusion probabilities: groupPIP denotes the probability that each cluster enters the model; condPIP ranks individual metabolites within clusters.

The bidirectional WQS model identified two mixture indices with opposite directions of effect (Figure 3a). After covariate adjustment, a one-unit increase in the positive index was associated with 25% higher odds of frailty (OR = 1.25, 95% CI 1.17–1.33), whereas a one-unit increase in the negative index corresponded to 17% lower odds (OR = 0.83, 95% CI 0.75–0.91).

In the positive index, the largest weights were assigned to DHBMA (22%) and HPMMA (21%), followed by CEMA (13%) and MA (8%). 3,4-MHA, CYMA and 2-HPMA each accounted for 5–7%, and no other metabolite exceeded 5%.

The negative index was led by SBMA (17%), AAMA (12%) and 2-MHA (10%). ATCA, MHBMA3 and 3-HPMA contributed 8–10%, while every remaining metabolite contributed less than 6%. In a sensitivity analysis using LASSO-selected metabolites within the WQS framework, the results remained consistent with those from the primary WQS model, further supporting the robustness of our findings (Supplementary Figure 4).

To confirm robustness, we ran a quantile g-computation analysis, which showed no overall association (OR = 1.02, 95% CI 0.86–1.21; *p* = 0.81); component weights appear in Supplementary Figure 5.

#### 3.3.2 BKMR analysis

To address multicollinearity and pinpoint influential VOCms, we ran 10-fold cross-validated LASSO logistic regression (Supplementary Figures 6, 7). Choosing the penalty at one standard error above the minimum deviance produced a parsimonious model with nine metabolites—four positively and five negatively related to frailty (Supplementary Table 4). These coefficient signs matched the qgcomp weights, supporting the positive- versus negative-grouping adopted for the BKMR analyses.

We then fitted three separate BKMR models to explore the overall and direction-specific mixture effects of these metabolites on frailty. For the overall BKMR model (Figure 3b), the estimated exposure–frailty relationship rises across quantiles, but its 95% confidence intervals consistently include zero, indicating no statistically significant association. In contrast, the positive-group BKMR curve (Figure 3c) is bidirectional: frailty risk is lower at the lowest quartiles, flattens near the 50th percentile, and then increases steadily at higher exposure levels. The negatively associated group showed a slight downward trend, but the overall effect was minimal and not statistically significant (Supplementary Figure 8). Figure 3d presents the hierarchical BKMR variable selection, identifying two metabolite clusters with groupPIPs of 0.999 and 1.000. In the first cluster, only ATCA showed a strong conditional inclusion probability (condPIP = 0.98), whereas 2MHA, SBMA, 2HPMA, and PGA were all ≤ 0.02. In the second cluster, DHBMA dominated (condPIP = 0.892), with MHBMA3 (0.09) and HPMMA (0.017) contributing modestly and CEMA effectively excluded (condPIP = 0.001). Supplementary Figure 9 shows that DHBMA is positively associated with frailty, as its 75th-percentile credible interval lies entirely above zero, whereas ATCA is inversely associated, with its 75th-percentile interval entirely below zero. All other metabolites have intervals crossing zero at every percentile, indicating no clear link to frailty. Supplementary Figure 10 shows marginal BKMR curves for each VOCm (others fixed at their median). DHBMA rises linearly with frailty across its full range. MHBMA3 and ATCA both peak at intermediate exposures, forming inverted-U shapes. All other metabolites hug the null line with 95% credible intervals crossing zero, indicating negligible individual effects.

### 3.4 Subgroup analysis

In stratified WQS models (Supplementary Figure 11), the positive mixture index was significant in all strata—men (OR = 1.13, 95% CI 1.04–1.23), women (OR = 1.27, 95% CI 1.11–1.46), adults < 65 y (OR = 1.19, 95% CI 1.03–1.37) and adults ≥ 65 y (OR = 1.35, 95% CI 1.09–1.68). The metabolite composition of the positive index varied by subgroup: MHBMA3 contributed most in men (18%), HPMMA in women (16%), while DHBMA dominated both age groups—accounting for 19% of the weight in adults < 65 y and 15% in adults ≥ 65 y. For the negative index, significant associations appeared only in women (OR = 0.80, 95% CI 0.71–0.90) and adults < 65 y (OR = 0.87, 95% CI 0.76–0.97). PGA carried the largest negative weight in both strata—14% in women and 15% in younger adults. Subgroup BKMR and qgcomp findings (Supplementary Figures 12, 13) were consistent with the unstratified analyses. In stratified analyses by smoking status, WQS and BKMR models showed stronger associations among current smokers, whereas associations in former and never smokers were weaker (Supplementary Figure 14).

### 3.5 Exploratory ratio analysis

As an exploratory analysis, we further examined the urinary DHBMA/SBMA ratio in relation to frailty (Supplementary Figure 15). Higher DHBMA/SBMA ratios were significantly associated with increased odds of frailty (OR = 1.54, 95% CI: 1.07–2.22, *p* = 0.025). When categorized into quartiles, individuals in the highest quartile (Q4) had significantly greater frailty risk compared with Q1 (OR = 1.52, 95% CI: 1.07–2.17, *p* = 0.026). A significant trend across quartiles was observed (p for trend = 0.014).

### 3.6 Mediated analysis

Table 3 summarizes the mediation of the VOCm–frailty association by oxidative-stress markers. For DHBMA, mediation via GGT, bilirubin, and albumin accounted for 3.69, 5.41, and 11.56% of the total effect, respectively, with the direct effect remaining predominant. For CEMA, bilirubin and albumin mediated 13.90 and 15.44%, respectively, while GGT mediation was negligible. MHBMA3 showed a 20.29% mediated share through bilirubin, with no significant mediation by GGT or albumin. For HPMMA, only bilirubin exhibited a significant indirect effect (12.00%). In addition, the oxidative balance score (OBS) significantly mediated the association between DHBMA and frailty (indirect effect = 0.00168, *p* = 0.044), accounting for 1.76% of the total effect, whereas no significant mediation effects of OBS were observed for other metabolites. Regarding inflammation, hs-CRP showed a significant mediation effect for CEMA (indirect effect = 0.0116, *p* = 0.046), explaining 9.21% of the total effect. However, mediation via hs-CRP was not observed for DHBMA, MHBMA3, or HPMMA. Given that hs-CRP measurements were only available in the 2015–2018 NHANES cycles, these findings should be interpreted with caution. Taken together, Table 3 presents only the statistically significant mediation pathways, while the full set of mediation results is provided in Supplementary Table 5.

**Table 3:** Significant mediation analysis between VOCms and frailty.

Result	VOCms	Mediator	Indirect effect	Direct effect	Total effect	Mediation proportions
			(*p*-value)	(*p*-value)	(*p*-value)	
Frailty	DHBMA	Gamma glutamyl transferase	0.004109 (0.042)	0.107177 (< 0.001)	0.111286 (< 0.001)	3.69%
		Bilirubin	0.006017 (0.012)	0.105181 (< 0.001)	0.111198 (< 0.001)	5.41%
		Albumin	0.01303 (0.01)	0.09966 (< 0.001)	0.11269 (< 0.001)	11.56%
		Dietary Antioxidant/Oxidant Balance Scores(OBS)	0.00168 (0.044)	0.0936 (<0.001)	0.0953 (<0.001)	1.76%
	CEMA	Bilirubin	0.01133 (< 0.001)	0.07020 (0.024)	0.08154 (0.01)	13.90%
		Albumin	0.01279 (0.008)	0.07005 (0.016)	0.08283 (0.004)	15.44%
		hs-CRP	0.011578 (0.046)	0.114155 (<0.001)	0.125733 (<0.001)	9.21%
	MHBMA3	Bilirubin	0.01599 (< 0.001)	0.06282 (0.024)	0.07881 (0.008)	20.29%
	HPMMA	Bilirubin	0.01000 (< 0.001)	0.07111 (0.008)	0.08111 (0.0006)	12.00%

## 4 Discussion

Our results can be distilled into four inter-locking observations that together delineate the full exposure-to-outcome continuum. First, in single-metabolite analyses we observed consistent, exposure-dependent associations for four urinary VOC metabolites—DHBMA, CEMA, HPMMA, and MHBMA3. Modeled as log₁₀-transformed continuous variables, each metabolite was positively related to frailty, with fully adjusted odds ratios (ORs) ranging from 1.67 to 2.59 per one-unit increase in concentration. When the same metabolites were categorized into quartiles, participants in the highest quartile (Q4) had 1.65- to 1.97-fold higher odds of frailty than those in the lowest quartile (Q1), reaffirming a monotonic, dose-responsive pattern. Second, when the 16 metabolites were treated as a mixture in two-directional weighted-quantile-sum (WQS) regression, we observed a clear bidirectional pattern: a one-unit increase in the positive-direction index was associated with a 25% increase in frailty odds, whereas a one-unit increase in the negative-direction index corresponded to a 17% decrease. Sex- and age-stratified WQS models showed that this risk-increasing (positive) index remained significant only in men and in adults ≥ 65 y; within that index, DHBMA dominated the weights in men (18%), whereas HPMMA led in women (16%), signaling sex-specific drivers. Third, Bayesian kernel machine regression (BKMR) built on LASSO-selected positive and negative groups corroborated these findings: the positive group displayed a monotonic, upward exposure–response curve, while the negative group showed no consistent relationship, reinforcing the notion that risk is concentrated in a small subset of metabolites. Fourth, causal-mediation analysis indicated that oxidative-stress markers—particularly albumin and bilirubin—mediated 5–20% of the associations for the four key metabolites, implicating oxidative imbalance as a partial pathway. Beyond conventional hepatic oxidative stress markers, we incorporated OBS and hs-CRP to strengthen the biological plausibility of the pathway interpretation. OBS provided partial evidence of oxidative imbalance mediation for DHBMA, while hs-CRP mediated the effect of CEMA on frailty. Notably, hs-CRP results were limited to NHANES 2015–2018, which may restrict generalizability and attenuate statistical power. A robustness check using quantile g-computation (qgcomp) produced a null overall mixture estimate.

Urinary mercapturic acids are increasingly recognized as sensitive sentinels of systemic VOC exposure. Earlier NHANES studies linked higher VOCm burdens to hypertension, metabolic syndrome and sarcopenia, suggesting that solvent exposure affects multiple organ systems (4, 19, 35). Recent evidence has moved further toward functional-ageing outcomes. A multi-country analysis of community-dwelling older adults reported that reliance on unclean cooking fuels—an important source of indoor VOCs—was associated with slower gait speed and impaired balance, while a decade-long rural Chinese panel reached similar conclusions for mobility and quality-of-life indicators (36, 37). Experimental work echoes these observations: in mice, low-dose mixtures of butadiene- and acrolein-derived metabolites reduced skeletal-muscle mass and elevated reactive-oxygen species, whereas in cultured myotubes they disrupted mitochondrial membrane potential, damaged mtDNA and depleted glutathione (38, 39). Taken together, these human and mechanistic data portray VOCs as multi-system stressors that erode cardiovascular, metabolic and musculoskeletal reserves—the core physiological domains captured by frailty indices. Our finding that four metabolites (DHBMA, CEMA, HPMMA and MHBMA3) are positively associated with frailty in a nationally representative U.S. cohort therefore extends VOC research from organ-specific endpoints to an integrated ageing metric, underscoring the potential public-health value of stricter ambient and indoor VOC control.

The risk-increasing component of the mixture is driven by four metabolites—DHBMA, CEMA, HPMMA, and MHBMA3—whose epoxide or aldehyde groups readily deplete glutathione and initiate oxidative-stress cascades (40, 41). Sex-stratified WQS weights uncovered distinct lead compounds: in men, MHBMA3 (a metabolite of 1,3-butadiene) carried the greatest weight, whereas in women HPMMA (a metabolite of crotonaldehyde) predominated. Such differences are biologically plausible, given sex-specific patterns in glutathione-S-transferase expression and adipose storage that can modulate the internal dose of lipophilic VOCs (42, 43). Age further shaped the mixture profile. Among adults younger than 65 years, both the positive and the negative WQS indices remained significant. The inverse (negative) index—anchored by ATCA and 2-MHA—most likely reflects rapid metabolic clearance: efficient detoxification can elevate urinary metabolite concentrations without increasing true tissue burden. A short-lived hormetic response may also be involved; very low VOC doses have been shown to transiently activate Nrf2-dependent antioxidant pathways (44, 45), and the BKMR curve for ATCA exhibits an inverted-U pattern consistent with this mechanism. In contrast, the negative index disappeared in adults aged 65 years or older, a finding that dovetails with well-documented age-related declines in glutathione synthesis and renal excretory capacity. Under these conditions, elevated urinary VOCm levels are more likely to signify genuine internal exposure rather than swift elimination. BKMR likewise detected no protective signal in either age stratum, reinforcing the view that the apparent benefit seen in younger adults arises from detoxification kinetics or transient hormesis rather than inherently benign chemistry. Collectively, these subgroup patterns highlight biotransformation capacity and redox reserve as critical modifiers of the VOC-frailty relationship. Our WQS regression analyses identified significant mixture effects in both the positive and negative directions. Although this may initially appear counterintuitive, it reflects the internal heterogeneity of the VOC mixture. Specifically, certain metabolites demonstrated positive associations with frailty risk, while others showed inverse associations, resulting in bidirectional mixture effects when modeled separately. For instance, the exploratory ratio analysis revealed that a higher DHBMA/SBMA concentration ratio was associated with increased frailty risk. These findings indicate that the predominance of DHBMA over SBMA may elevate the likelihood of frailty, pointing to potential synergistic and antagonistic interactions within the VOC mixture. This ratio-based approach provides an additional perspective for capturing the complexity of co-exposures, complementing insights from single-pollutant models. Taken together, these findings indicate that VOC exposures may not act uniformly but instead involve both risk-enhancing and potentially protective components, highlighting the complexity of real-world chemical mixtures.

The main risk metabolites—DHBMA, CEMA, HPMMA, and MHBMA3—originate from VOCs (1,3-butadiene, acrylonitrile, crotonaldehyde) that form reactive epoxides or aldehydes, deplete glutathione, and generate ROS (41). Lab work supports this: butadiene epoxide boosts malondialdehyde and disrupts mitochondrial potential, while acrolein derivatives give only a brief Nrf2 surge before antioxidants are overwhelmed (46, 47). Although animal studies provide important mechanistic insights, caution is needed when extrapolating these findings to humans, as not all pathways directly translate. Our mediation analysis echoes this biology. The systemic redox markers bilirubin and albumin jointly accounted for 5–20% of the frailty association for each metabolite (greatest for MHBMA3), whereas the hepatic enzyme *γ*-glutamyl-transferase contributed little, pointing to generalized rather than liver-specific oxidative stress. Because most of the total effect remained direct, additional pathways—such as endocrine disruption, low-grade inflammation, or mitochondrial dysfunction—are likely involved. Taken together, the partial statistical mediation and convergent toxicological evidence render oxidative imbalance a credible, although not yet definitive, mechanistic bridge between VOC exposure, as indexed by urinary metabolites, and frailty.

Smoking is a well-recognized source of VOC exposure. In our stratified analyses, associations between urinary VOC metabolites and frailty were more evident among current smokers, while results were less consistent in former and never smokers. This suggests that smoking status influences the detectability of VOC–frailty relationships. Nevertheless, several of the key VOCs identified, including 1,3-butadiene, crotonaldehyde, and xylene, also derive from environmental and occupational sources unrelated to tobacco smoke. Thus, while smoking remains an important contributor, it does not fully explain the observed associations. Residual confounding by smoking-related exposures cannot be entirely excluded and should be considered when interpreting our findings.

This study is the first to relate urinary VOCm mixtures to frailty in a nationally representative sample of U.S. adults. By integrating three complementary mixture approaches—direction-specific WQS, grouped BKMR, and qgcomp—together with oxidative-stress mediation, we were able to triangulate risk estimates and pinpoint sex- and age-specific drivers. The convergent signal across WQS and BKMR highlights a small subset of metabolites (DHBMA, CEMA, HPMMA, MHBMA3) as principal hazards, with mediation analysis indicating that systemic oxidative imbalance could represent one possible mechanism underlying the observed associations. These results reinforce the notion that VOC control could promote healthier ageing trajectories.

Several caveats temper these conclusions. First, the NHANES design is cross-sectional; directionality and causality cannot be established. Second, exposure was assessed from a single spot-urine sample, which is susceptible to within-person variability and may misclassify long-term exposure—bias that would generally attenuate associations. Third, qgcomp yielded a null overall estimate; this likely reflects its lower statistical power when a mixture contains components with opposing effects, but it nonetheless underlines the need for caution in interpreting borderline associations. Fourth, residual confounding is possible, and sample sizes for sex- and age-stratified analyses were modest, limiting precision. Finally, NHANES is representative of the U.S. population; since VOC sources and exposure levels may differ across countries, the generalizability of our findings to other populations should be interpreted with caution.

Future work should couple repeated urinary and blood measurements with personal air monitoring, apply high-resolution exposomic and multi-omics panels, and follow participants longitudinally to confirm temporality, delineate additional pathways—such as endocrine or mitochondrial disruption—and test whether targeted VOC-reduction strategies can meaningfully delay frailty onset.

## 5 Conclusion

Urinary VOCm mixtures—driven by metabolites DHBMA, CEMA, HPMMA, and MHBMA3—are linked to greater frailty, especially in men and adults ≥ 65 y. Oxidative stress mediates part of this risk, yet most effects remain direct. Controlling environmentally relevant VOC exposure could support healthier ageing; longitudinal confirmation is warranted.

## Data Availability

The original contributions presented in the study are included in the article/Supplementary material, further inquiries can be directed to the corresponding author.
