# Supplementary material for: Volatile organic compounds exposure associated with frailty in United States adults from NHANES 2011–2018
Source: Front Public Health. 2025 Sep 16;13:1655214. doi: 10.3389/fpubh.2025.1655214 (PMC12479300; doi:10.3389/fpubh.2025.1655214)
Supplement: Supplementary file 1 [file Table_1.DOCX]

**Supplementary** **Table 1.** Variables in the 48-item frailty index and their respective scorings.

| Variable | Scoring |
| --- | --- |
| Cognition |  |
| 1. Experience confusion/memory problems | Yes = 1, No = 0 |
| Dependence |  |
| 2. Managing money | Difficulty = 1, No Difficulty = 0 |
| 3. Stooping, crouching, kneeling | Difficulty = 1, No Difficulty = 0 |
| 4. Lifting or carrying | Difficulty = 1, No Difficulty = 0 |
| 5. House chore | Difficulty = 1, No Difficulty = 0 |
| 6. Preparing meals | Difficulty = 1, No Difficulty = 0 |
| 7. Standing up from armless chair | Difficulty = 1, No Difficulty = 0 |
| 8. Getting in and out of bed difficulty | Difficulty = 1, No Difficulty = 0 |
| 9. Using fork, knife, drinking from cup | Difficulty = 1, No Difficulty = 0 |
| 10. Dressing yourself | Difficulty = 1, No Difficulty = 0 |
| 11. Standing for long periods difficulty | Difficulty = 1, No Difficulty = 0 |
| 12. Grasp/holding small objects | Difficulty = 1, No Difficulty = 0 |
| 13. Attending social event | Difficulty = 1, No Difficulty = 0 |
| 14. Push or pull large objects | Difficulty = 1, No Difficulty = 0 |
| 15. Walking for a quarter mile difficulty | Difficulty = 1, No Difficulty = 0 |
| 16. Walking up 10 steps difficulty | Difficulty = 1, No Difficulty = 0 |
| Depressive Symptoms |  |
| 17. Have little interest in doing things | Nearly every day = 1, More than half the days = 0.66, Several days = 0.33, Not at all = 0 |
| 18. Feeling down, depressed, or hopeless | Nearly every day = 1, More than half the days = 0.66, Several days = 0.33, Not at all = 0 |
| 19. Trouble sleeping or sleeping too much | Nearly every day = 1, More than half the days = 0.66, Several days = 0.33, Not at all = 0 |
| 20. Feeling tired or having little energy | Nearly every day = 1, More than half the days = 0.66, Several days = 0.33, Not at all = 0 |
| 21. Poor appetite or overeating | Nearly every day = 1, More than half the days = 0.66, Several days = 0.33, Not at all = 0 |
| 22. Feeling bad about yourself | Nearly every day = 1, More than half the days = 0.66, Several days = 0.33, Not at all = 0 |
| 23. Trouble concentrating on things | Nearly every day = 1, More than half the days = 0.66, Several days = 0.33, Not at all = 0 |
| Comorbidities |  |
| 24. Arthritis | Yes = 1, Suspect = 0.5, No = 0 |
| 25. Thyroid problems | Yes = 1, Suspect = 0.5, No = 0 |
| 26. Chronic bronchitis | Yes = 1, Suspect = 0.5, No = 0 |
| 27. Cancer | Yes = 1, Suspect = 0.5, No = 0 |
| 28. Congestive heart failure | Yes = 1, Suspect = 0.5, No = 0 |
| 29. Coronary heart disease | Yes = 1, Suspect = 0.5, No = 0 |
| 30. Angina | Yes = 1, Suspect = 0.5, No = 0 |
| 31. Heart attack | Yes = 1, Suspect = 0.5, No = 0 |
| 32. Stroke | Yes = 1, Suspect = 0.5, No = 0 |
| 33. Hypertension | Yes = 1, Suspect = 0.5, No = 0 |
| 34. Diabetes | Yes = 1, Suspect = 0.5, No = 0 |
| 35. weak/failing kidneys | Yes = 1, Suspect = 0.5, No = 0 |
| 36. Urinary Leakage | Yes = 1, Suspect = 0.5, No = 0 |
| Hospital Utilization and Access to Care |  |
| 37. Self-rated health | Fair, poor = 1, Excellent, Very good, good = 0 |
| 38. Health now compared with 1 year ago | Worse = 1, About the same, better = 0 |
| 39. Overnight hospital patient in past year | Yes = 1, No = 0 |
| 40. Frequency of health care use during past year | None = 0, 1-3 = 0.5, More than 3 = 1 |
| 41. Number of prescribed medications | None = 0, 1-4 = 0.5, 5 and more = 1 |
| Anthropometry and Laboratory Values |  |
| 42. BMI | <18.5, ≥30 = 1; 25- <30 = 0.5; 18.5-25 = 0 |
| 43. Glycohemoglobin (%) | 0-5.7 = 0, >5.7 = 1 |
| 44. Red blood cell count (million cells/μL) | M: 4.7-6.1 = 0, Other = 1; F: 4.2-5.4 = 0, Other = 1 |
| 45. Hemoglobin (g/dL) | M: 13.5-18 = 0, Other = 1; F: 12-16 = 0, Other = 1 |
| 46. Red cell distribution width (%) | 11.6-14.6 = 0, Other = 1 |
| 47. Lymphocyte percent (%) | 20-40 = 0, Other = 1 |
| 48. Segmented neutrophils percent (%) | 40-80 = 0, Other = 1 |

BMI, Body mass index; M, Male; F, Female.

**Supplementary Figure 2.** Scheme for the assignment of OBS.

| **OBS components** | **Property** | **Male** | | | **Female** | | |
| --- | --- | --- | --- | --- | --- | --- | --- |
|  |  | **0** | **1** | **2** | **0** | **1** | **2** |
| **Dietary OBS** | | | | | | | |
| Dietary fiber (g/d) | A | <12.10 | 12.10–20.50 | ≥20.50 | <9.80 | 9.80–16.20 | ≥16.20 |
| Carotene (RE/d) | A | <33. 81 | 33.81–108.00 | ≥108.00 | <33.26 | 33.26–132.30 | ≥132.30 |
| Riboflavin (mg/d) | A | <1.72 | 1.72–2.66 | ≥2.66 | <1.30 | 1.30–2.01 | ≥2.01 |
| Niacin (mg/d) | A | <21.82 | 21.82–33.78 | ≥33.78 | <15.06 | 15.06–23.13 | ≥23.13 |
| Total folate (mcg/d) | A | <318.00 | 318.00–499.10 | ≥499.10 | <236.00 | 236.00–378.00 | ≥378.00 |
| Calcium (mg/d) | A | <661.00 | 661.00–1123.10 | ≥1123.10 | <544.27 | 544.27–937.00 | ≥937.00 |
| Zinc (mg/d) | A | <9.43 | 9.43–14.96 | ≥14.96 | <6.67 | 6.67–10.65 | ≥10.65 |
| Magnesium (mg/d) | A | <253.00 | 253.00–375.00 | ≥375.00 | <195.00 | 195.00–288.00 | ≥288.00 |
| Copper (mg/d) | A | <1.07 | 1.07–1.59 | ≥1.59 | <0.84 | 0.84–1.25 | ≥1.25 |
| Selenium (mcg/d) | A | <98.80 | 98.80–150.81 | ≥150.81 | <69.60 | 69.60–106.40 | ≥106.40 |
| Iron (mg/d) | P | ≥19.16 | 12.51–19.16 | <12.51 | ≥14.33 | 9.16–14.33 | <9.16 |
| Total fat (g/d) | P | ≥108.70 | 69.97–108.70 | <69.97 | ≥79.48 | 51.71–79.48 | <51.71 |
| Vitamin B6 (mg/d) | A | <1.63 | 1.63–2.59 | ≥2.59 | <1.15 | 1.15–1.85 | ≥1.85 |
| Vitamin B12 (mcg/d) | A | <3.20 | 3.20–6.31 | ≥6.31 | <2.07 | 2.07–4.26 | ≥4.26 |
| Vitamin C (mg/d) | A | <31.00 | 31.00–99.11 | ≥99.11 | <28.03 | 28.03–84.01 | ≥84.01 |
| Vitamin E (ATE) (mg/d) | A | <5.57 | 5.57–9.43 | ≥9.43 | <4.45 | 4.45–7.53 | ≥7.53 |
| **Lifestyle OBS** | | | | | | | |
| Physical activity (MET-minute/week) | A | <400.00 | 400.00–1680.00 | ≥1680.00 | <409.50 | 409.50–1440.00 | ≥1440.00 |
| Body mass index (kg/m^2^) | P | ≥29.10 | 25.30–29.10 | <25.30 | ≥29.59 | 24.19–29.59 | <24.19 |
| Alcohol (g/d) | P | ≥30 | 0–30 | None | ≥15 | 0–15 | None |
| Cotinine (ng/mL) | P | ≥8.38 | 0.03–8.38 | <0.03 | ≥0.13 | 0.02–0.13 | <0.02 |

OBS: oxidative balance score; A: antioxidant; P: prooxidant; RE: retinol equivalent; ATE: alpha-tocopherol equivalent; MET: metabolic equivalent.

**Supplementary Table 3.**Baseline characteristics of study participants after k-nearest neighbors (KNN) imputation

| Characteristics | Total | Non-frailty | Frailty | P-value |
| --- | --- | --- | --- | --- |
| No. of participants | 2715 | 1332 | 1383 |  |
| Age (years) | 64(56-72) | 64(60-71) | 64(54-74) | 0.637 |
| Sex, % |  |  |  | <0.001 |
| Female | 1322(48.69%) | 556 (41.74%) | 766 (55.39%) |  |
| Male | 1393(51.31%) | 776 (58.26%) | 617 (44.61%) |  |
| Ethnicity, % |  |  |  | <0.001 |
| Mexican American | 300(11.05%) | 158 (11.86%) | 142 (10.27%) |  |
| Other Hispanic | 282(10.39%) | 140 (10.51%) | 142 (10.27%) |  |
| Non-Hispanic White | 1130(41.62%) | 532 (39.94%) | 598 (43.24%) |  |
| Non-Hispanic Black | 656(24.16%) | 298 (22.37%) | 358 (25.89%) |  |
| Non-Hispanic Asian | 245(9.02%) | 169 (12.69%) | 76 (5.50%) |  |
| Other Race | 102(3.76%) | 35 (2.63%) | 67 (4.84%) |  |
| Education level % |  |  |  | <0.001 |
| Less than high school | 363(13.37%) | 159 (11.94%) | 204 (14.75%) |  |
| High school or GED | 1058(38.97%) | 481 (36.11%) | 577 (41.72%) |  |
| Above high school | 1294(47.66%) | 692 (51.95%) | 602 (43.53%) |  |
| Physical activity |  |  |  | <0.001 |
| Never | 886(32.63%) | 320 (24.02%) | 566 (40.93%) |  |
| Moderate | 406(14.95%) | 186 (13.96%) | 220 (15.91%) |  |
| Vigorous | 1423(52.41%) | 826 (62.01%) | 597 (43.17%) |  |
| Smoking status |  |  |  | <0.001 |
| Never | 1274(46.92%) | 704 (52.85%) | 570 (41.21%) |  |
| Former | 569(20.96%) | 218 (16.37%) | 351 (25.38%) |  |
| Current | 872(32.12%) | 410 (30.78%) | 462 (33.41%) |  |
| Family income, % |  |  |  | <0.001 |
| Q1(<1.020) | 699(25.75%) | 254 (19.07%) | 445 (32.18%) |  |
| Q2(1.020–1.770) | 683(25.16%) | 306 (22.97%) | 377 (27.26%) |  |
| Q3(1.770–3.525) | 677(24.94%) | 354 (26.58%) | 323 (23.36%) |  |
| Q4(>3.525) | 656(24.16%) | 418 (31.38%) | 238 (17.21%) |  |
| Total energy intake(kcal) |  |  |  | 0.004 |
| Q1(<1362) | 661(24.35%) | 292 (21.92%) | 369 (26.68%) |  |
| Q2(1362–1812) | 688(25.34%) | 326 (24.47%) | 362 (26.17%) |  |
| Q3(1812–2355) | 694(25.56%) | 356 (26.73%) | 338 (24.44%) |  |
| Q4(>2355) | 672(24.75%) | 358 (26.88%) | 314 (22.70%) |  |

The continuous variables were presented as median (interquartile range, IQR), and the categorical variables were presented as number and percentages.

**Supplementary Figure 1.** Non-adjusted logistic regression associations of urinary VOC metabolites with frailty.

Forest plot displays survey-weighted odds ratios (ORs) and 95 % confidence intervals (horizontal bars) obtained from multivariable logistic regression models. The dashed vertical line marks the null value (OR = 1). Red squares highlight metabolites with statistically significant associations (two-sided p < 0.05); black squares indicate non-significant findings.


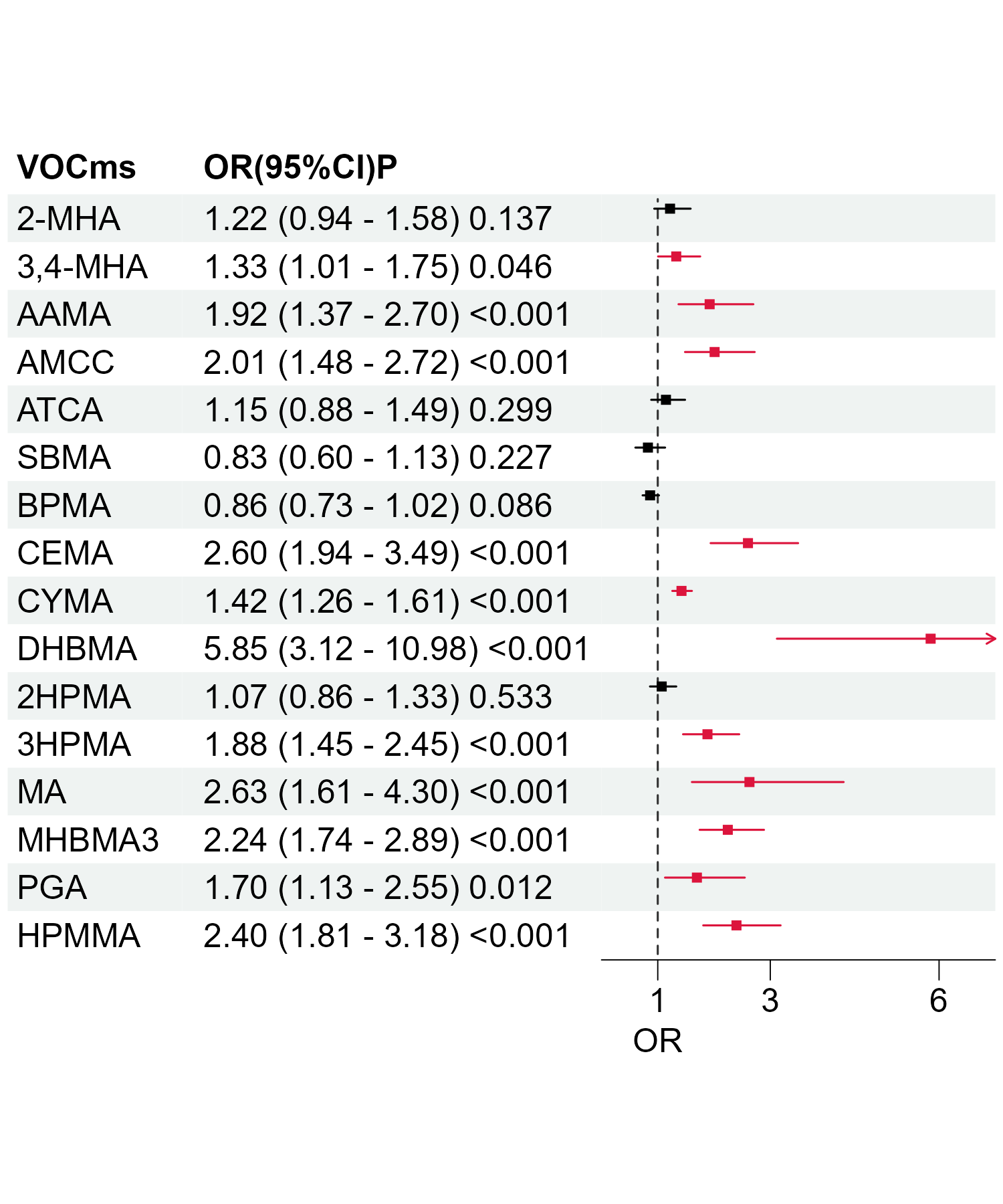


**Supplementary Figure 2.** Forest plot of multivariable logistic regression models examining the association between urinary VOC metabolites and frailty, additionally adjusted for BMI.

Forest plot displays survey-weighted odds ratios (ORs) and 95 % confidence intervals (horizontal bars) obtained from multivariable logistic regression models. Each model was adjusted for sex, age, educational attainment, race/ethnicity, poverty-income ratio, smoking status, physical activity, survey cycle, and total energy intake, with further adjustment for BMI. The dashed vertical line marks the null value (OR = 1). Red squares highlight metabolites with statistically significant associations (two-sided p < 0.05); black squares indicate non-significant findings.


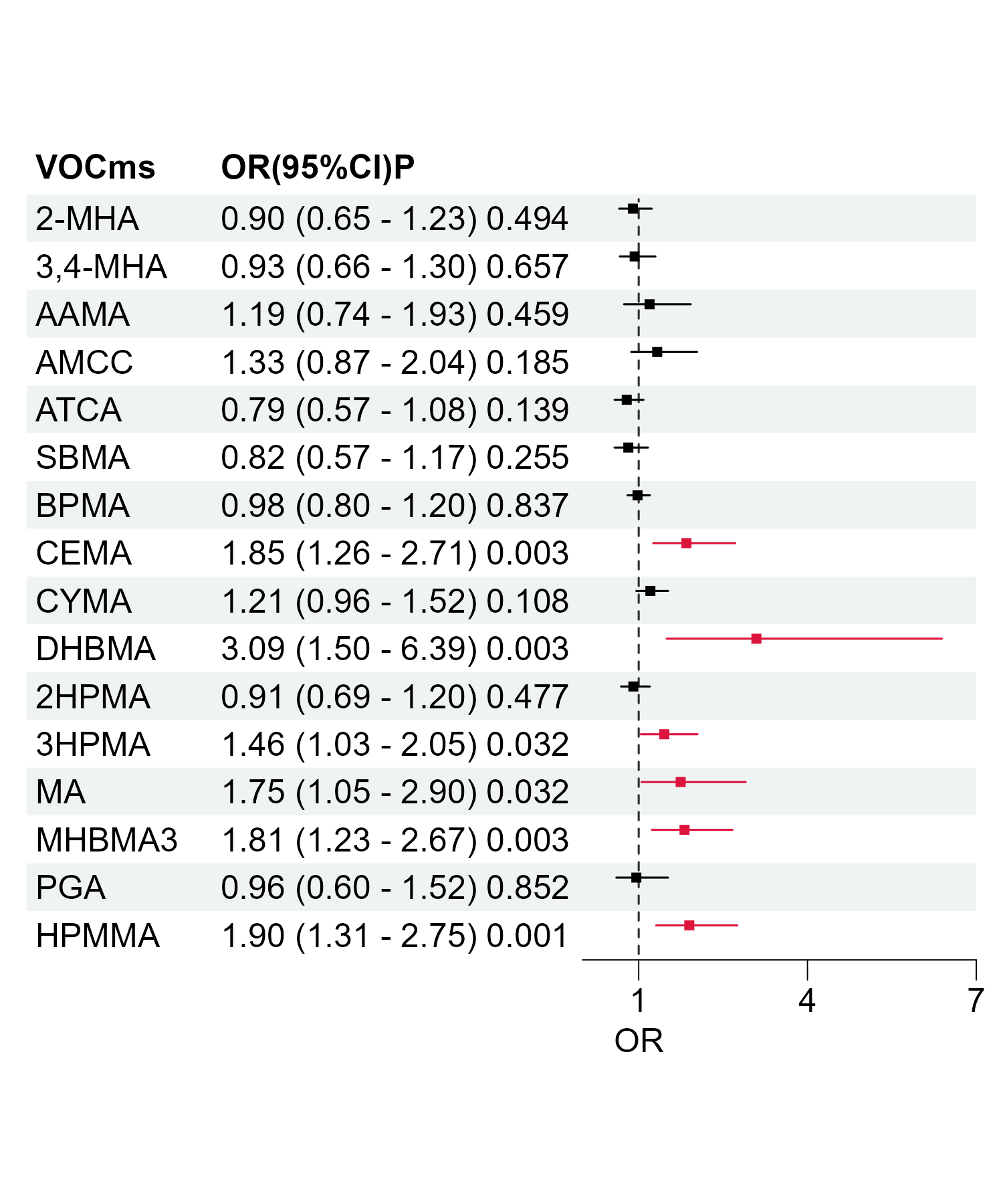


**Supplementary** **Figure 3.** Multivariable-adjusted odds ratios for frailty across quartiles of urinary mVOC metabolites.

Survey-weighted multivariable logistic-regression models were fitted for each metabolite. Concentrations were creatinine-adjusted, log-transformed, and divided into weighted quartiles; Quartile 1 (Q1) serves as the reference. Models adjust for sex, age, race/ethnicity, educational attainment, poverty-income ratio, smoking status, physical activity, total energy intake, and survey cycle, incorporating NHANES strata and primary sampling units. Points show odds ratios (ORs); horizontal bars give 95 % confidence intervals. The dashed vertical line marks the null (OR = 1). Red squares denote statistically significant associations (two-sided P < 0.05); black squares denote non-significant results. Cut-off values for each quartile appear in the left column.


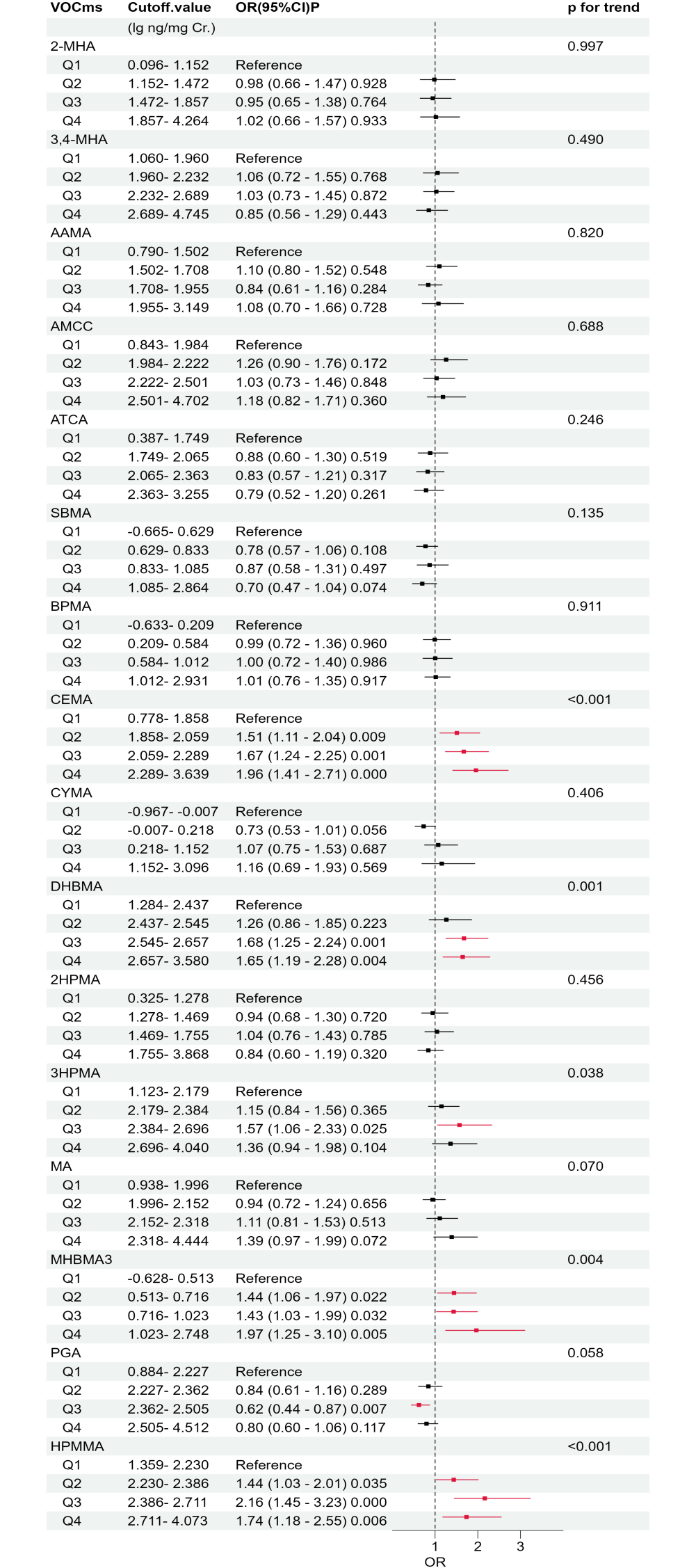


**Supplementary Figure 4.** WQS analysis with LASSO-selected metabolites.Bars to the right indicate positive (risk-enhancing) weights; bars to the left indicate negative (protective) weights. The red dashed line marks the equal-weight threshold (sum of weights = 1 in each direction). All models adjust for covariates as in the main analysis.


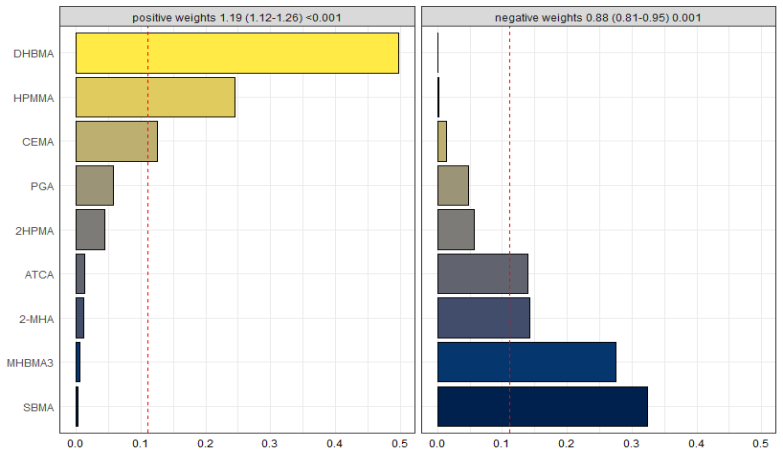


**Supplementary** **Figure 5**. Weights of mVOC components estimated by the qgcomp model.

Variable weights from the qgcomp model assessing the association between mVOC mixtures and frailty, adjusting for sex, age, educational level, race/ethnicity, poverty-income ratio, smoking status, physical activity, and total energy intake. Positive weights indicate harmful effects, while negative weights suggest protective effects. Weights are scaled to sum to 1 within each direction.


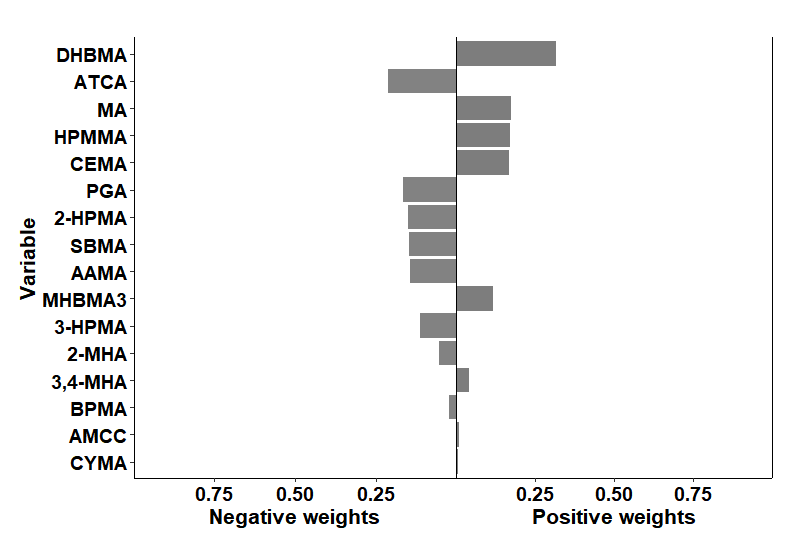


**Supplementary Figure 6.** LASSO model tuning plot for variable selection.

Ten-fold cross-validation was used to select the optimal penalty parameter (λ) in the LASSO regression. The red dots represent the mean binomial deviance for each value of log(λ), and the error bars represent ±1 standard error. The left dashed line indicates the λ with minimum deviance (λ_min), and the right dashed line indicates the largest λ within 1 standard error (λ_1se), typically used for a more parsimonious model.

**
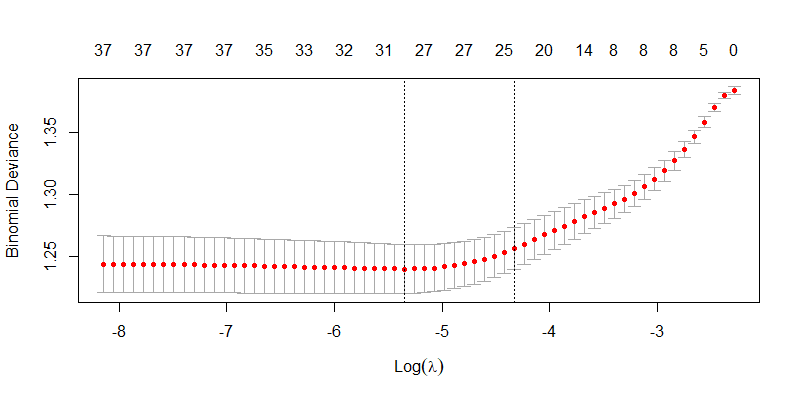
**

**Supplementary Figure 7.** LASSO coefficient profiles for urinary mVOC metabolites.

Each colored line represents the trajectory of a metabolite’s regression coefficient across a sequence of log(λ) values. As the penalty increases, coefficients shrink toward zero. The vertical dashed line marks the selected λ value at which key variables were retained for further analysis.


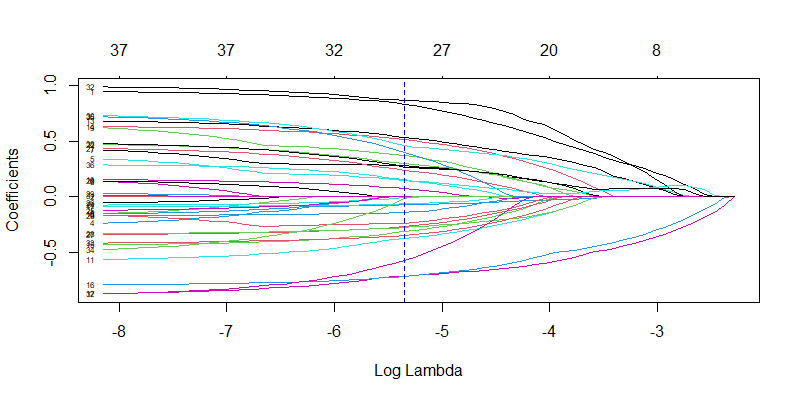


**Supplementary Table 4.**  LASSO-Selected mVOC Metabolites by Direction of Association

| Positive Group | Negative Group |
| --- | --- |
| CEMA | 2-MHA |
| DHBMA | ATCA |
| MHBMA3 | SBMA |
| HPMMA | 2HPMA |
|  | PGA |

**Supplementary Figure 8.** BKMR exposure–response curve for the negatively associated mVOC group.The curve plots the estimated change in frailty risk across deciles of the weighted mixture index for the five negatively associated metabolites (Group 1).


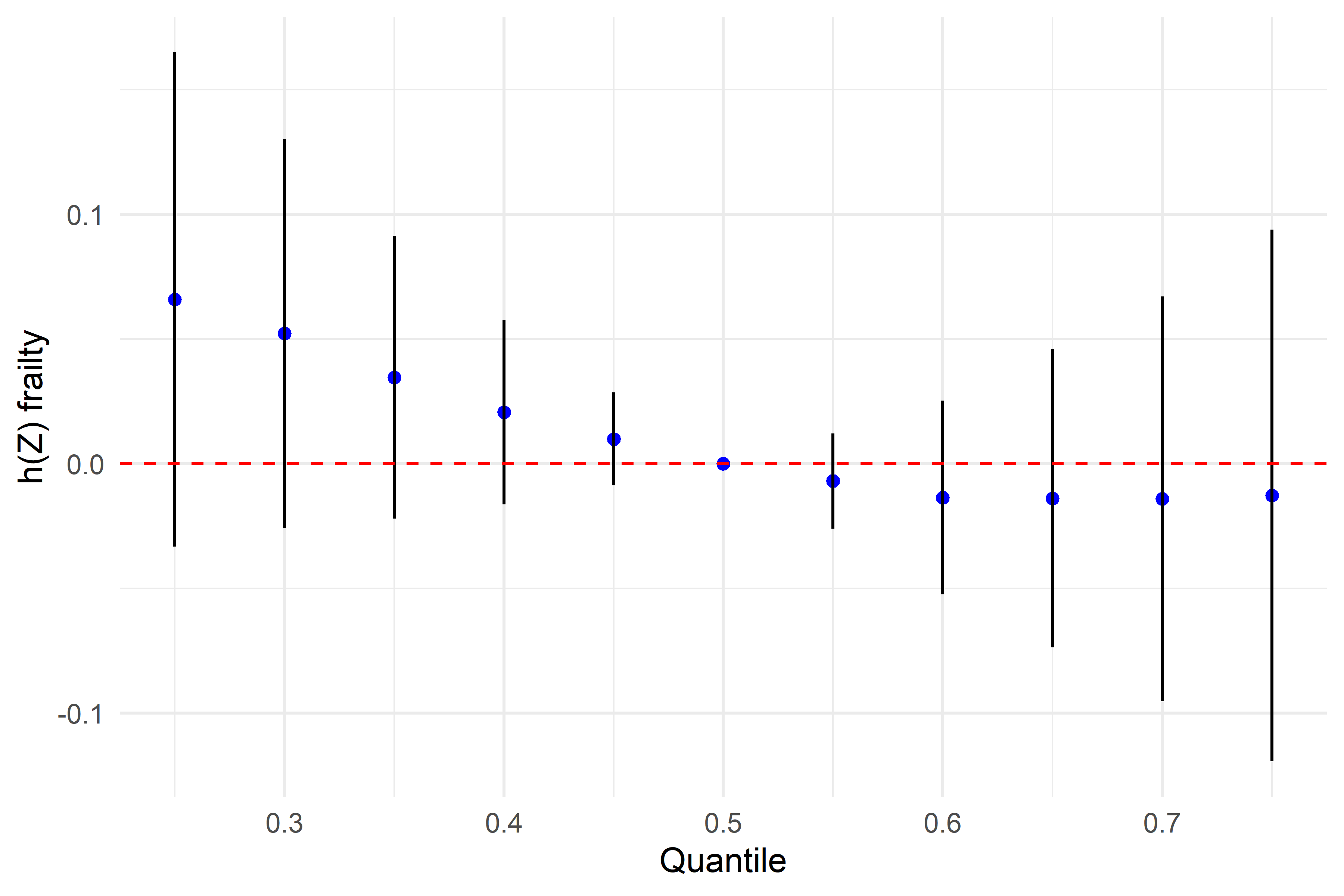


**Supplementary Figure 9.** Component-specific BKMR exposure–response estimates at fixed quantiles.Points show the estimated frailty effect (h(Z)) of each mVOC at the 25th (red), 50th (green), and 75th (blue) percentiles of the overall mixture index. Horizontal lines denote 95 % credible intervals. Metabolites are ordered by their median (50th-percentile) estimate, illustrating how each component’s contribution to frailty changes across low, mid, and high exposure levels.


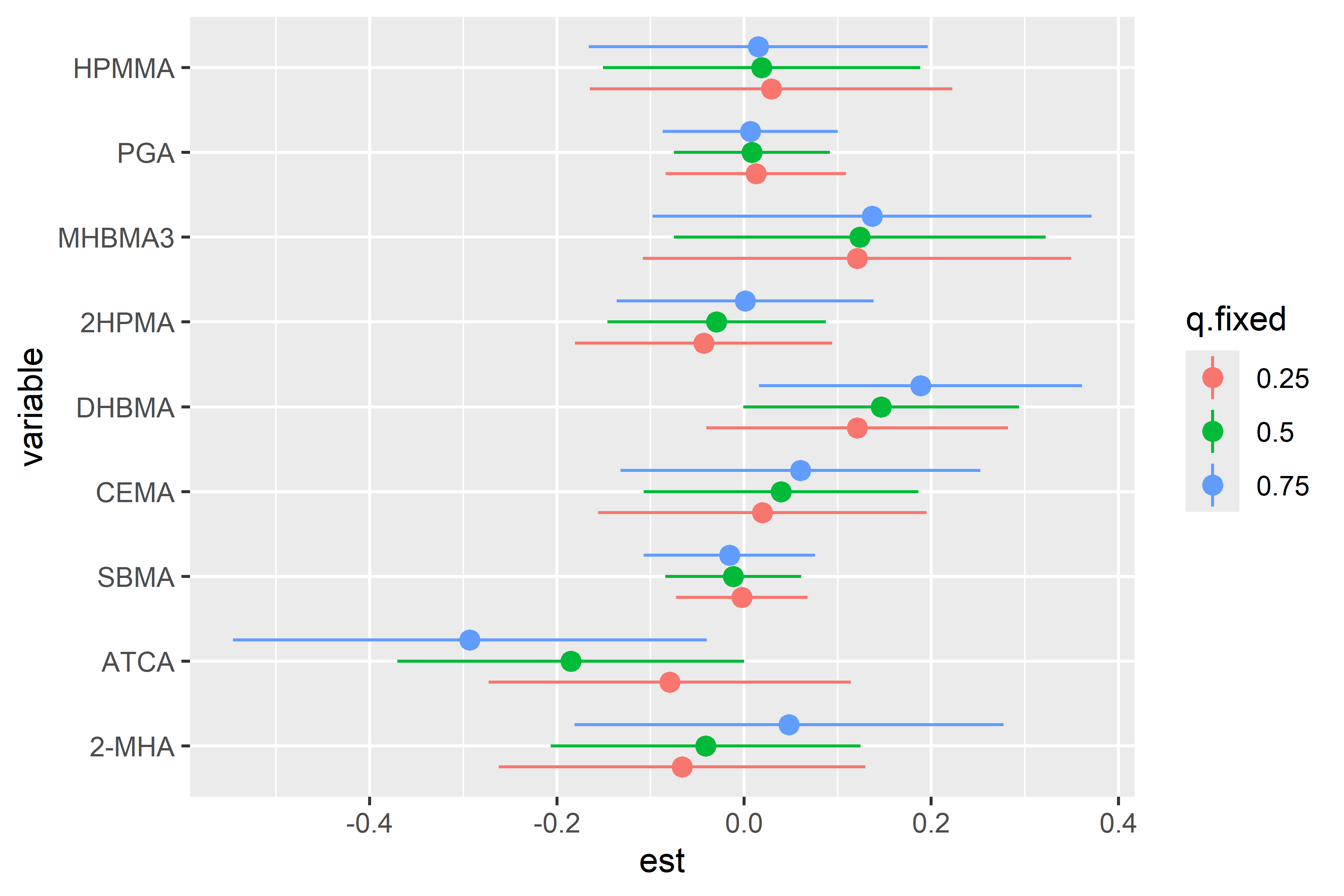


**Supplementary Figure 10.** Marginal BKMR exposure–response functions for individual mVOCs. Each panel shows the estimated frailty function, h(Z), for one mVOC across its standardized concentration (z), with all other exposures held at their median. The solid blue line represents the posterior mean h(Z) and the shaded gray area the 95 % credible interval.


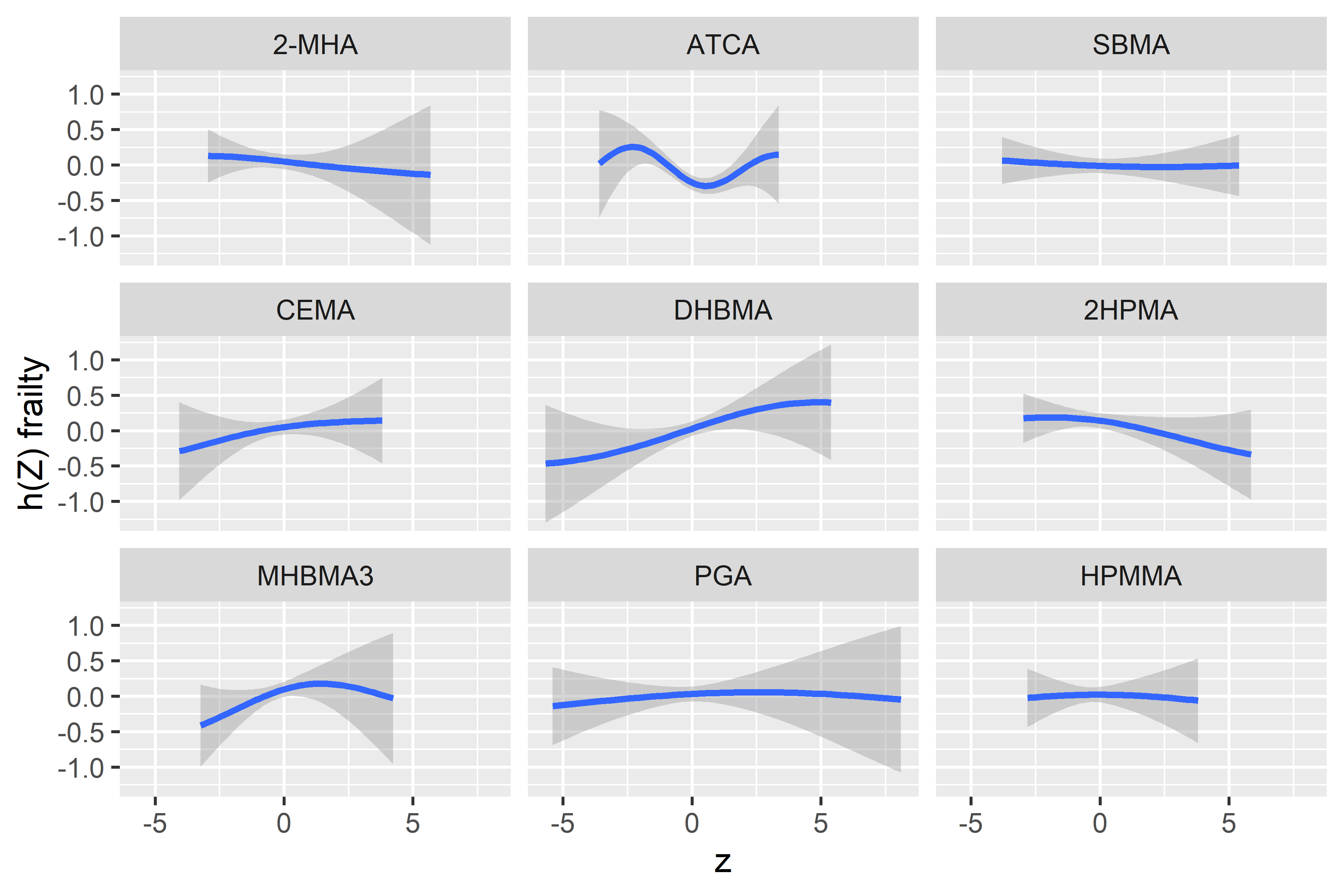


**Supplementary Figure 11.**  Two-directional WQS component weights stratified by sex and age. (a)Men; (b)Women; (c)Age ≥65; (d)Age <65. Bars to the right indicate positive (risk-enhancing) weights; bars to the left indicate negative (protective) weights. The red dashed line marks the equal-weight threshold (sum of weights = 1 in each direction). All models adjust for covariates as in the main analysis.

**Supplementary Figure 12.** BKMR exposure–response curves for mVOC mixtures by sex and age

Panels a–c depict results for men (a. overall mixture; b. positive group; c. negative group), panels d–f for women (d. overall mixture; e. positive group; f. negative group), panels g–i for participants aged ≥ 65 years (g. overall mixture; h. positive group; i. negative group), and panels j–l for those aged < 65 years (j. overall mixture; k. positive group; l. negative group). Each curve shows the posterior mean frailty function h(Z) across exposure quantiles with 95 % credible intervals (shaded), and the red dashed line marks the null effect; curves above this line indicate elevated frailty risk and those below indicate protective associations.

**Supplementary Figure 13.** Sex- and age-stratified qgcomp component weights for urinary mVOCs. (a)Men; (b)Women; (c)Age ≥65; (d)Age <65. Positive weights indicate metabolites linked to increased frailty odds; negative weights indicate protective associations. Bars are normalized so sums of positive and negative weights each equal one.

**Supplementary Figure 14.** Subgroup analyses of WQS and BKMR models stratified by smoking status.Panels a–c for WQS analyses stratified by smoking status (a. never smokers; b. former smokers; c. current smokers). Panels d–f for BKMR analyses among never smokers (d. overall mixture; e. positive group; f. negative group). Panels g–i for BKMR analyses among former smokers (g. overall mixture; h. positive group; i. negative group). Panels j–l for BKMR analyses among current smokers (j. overal mixture; k. positive group; l. negative group)

**Supplementary Figure 15.** Association between the urinary DHBMA/SBMA ratio and frailty

Survey-weighted logistic regression models showing the association between the urinary DHBMA/SBMA concentration ratio and frailty. The ratio was analyzed both as a continuous variable and in quartiles (Q1–Q4, with Q1 as the reference). Odds ratios (ORs) and 95% confidence intervals (CIs) are presented, adjusted for covariates including age, sex, race/ethnicity, education, poverty-income ratio, smoking status, physical activity, and total energy intake.


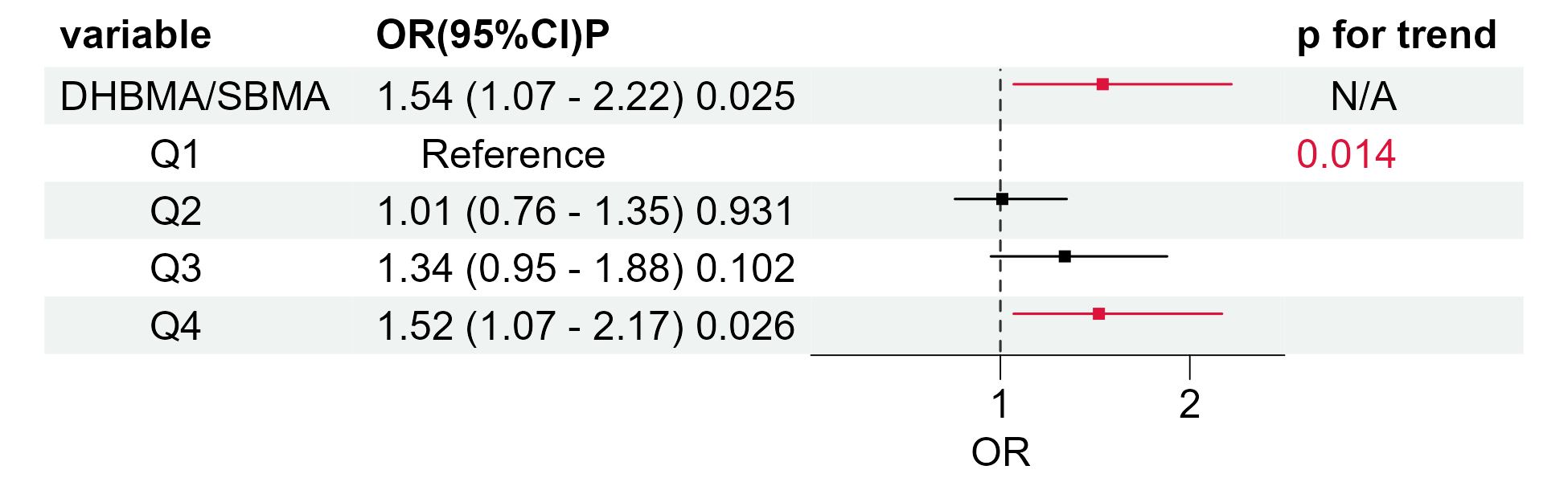


Supplementary Table 5. Full mediation analysis between VOCms and frailty

| **Result** | **VOCms** | **Mediator** | **Indirect effect** | **Direct effect** | **Total effect** | **Mediation proportions** |
| --- | --- | --- | --- | --- | --- | --- |
|  |  |  | **(**P-value) | **(**P-value) | **(**P-value) |  |
| Frailty | DHBMA | Gamma glutamyl transferase | 0.004109(0.042) | 0.107177(< 0.001) | 0.111286(< 0.001) | 3.69% |
|  |  | Bilirubin | 0.006017(0.012) | 0.105181(< 0.001) | 0.111198(< 0.001) | 5.41% |
|  |  | Albumin | 0.01303(0.01) | 0.09966(< 0.001) | 0.11269(< 0.001) | 11.56% |
|  |  | Dietary Antioxidant/Oxidant Balance Scores（OBS） | 0.00168（0.044） | 0.0936（<0.001) | 0.0953(<0.001) | 1.76% |
|  |  | hs-CRP | -0.01194(0.070) | 0.12179( < 0.001) | 0.10986(<0.001) | NA |
|  | CEMA | Gamma glutamyl transferase | 0.002646(0.204) | 0.080265(0.004) | 0.082910(0.004) | NA |
|  |  | Bilirubin | 0.01133(< 0.001) | 0.07020(0.024) | 0.08154(0.01) | 13.90% |
|  |  | Albumin | 0.01279(0.008) | 0.07005(0.016) | 0.08283(0.004) | 15.44% |
|  |  | Dietary Antioxidant/Oxidant Balance Scores（OBS） | 0.0001(0.936) | 0.0916(0.002) | 0.0917(0.002) | NA |
|  |  | hs-CRP | 0.011578(0.046) | 0.114155(<0.001) | 0.125733(<0.001) | 9.21% |
|  | MHBMA3 | Gamma glutamyl transferase | 0.000908(0.638) | 0.078698(0.006) | 0.079606( 0.004) | NA |
|  |  | Bilirubin | 0.01599(< 0.001) | 0.06282(0.024) | 0.07881( 0.008) | 20.29% |
|  |  | Albumin | 0.00113(0.824) | 0.07768(0.002) | 0.07882(<0.008) | NA |
|  |  | Dietary Antioxidant/Oxidant Balance Scores（OBS） | 0.00109(0.384) | 0.06638(0.008) | 0.06748(0.004) | NA |
|  |  | hs-CRP | -0.00320(0.59) | 0.15182(<0.001) | 0.14862(<0.001) | NA |
|  | HPMMA | Gamma glutamyl transferase | 0.002991(0.062) | 0.077101(0.002) | 0.080091(0.002) | NA |
|  |  | Bilirubin | 0.01000(< 0.001) | 0.07111(0.008) | 0.08111(0.0006) | 12.00% |
|  |  | Albumin | -0.00688(0.16) | 0.08643(< 0.001) | 0.07956(< 0.001) | NA |
|  |  | Dietary Antioxidant/Oxidant Balance Scores（OBS） | 0.00209(0.848) | 0.078101(0.004) | 0.078310(0.004) | NA |
|  |  | hs-CRP | -0.0115(0.062) | 0.1251(<0.001) | 0.1136(0.004) | NA |
